# A Randomized Controlled Trial of the Use of Oral Glucose with or without Gentle Facilitated Tucking of Infants during Neonatal Echocardiography

**DOI:** 10.1371/journal.pone.0141015

**Published:** 2015-10-23

**Authors:** Pascal M. Lavoie, Amelie Stritzke, Joseph Ting, Mohammad Jabr, Amish Jain, Eddie Kwan, Ela Chakkarapani, Paul Brooks, Rollin Brant, Patrick J. McNamara, Liisa Holsti

**Affiliations:** 1 Children’s & Women’s Health Centre of British Columbia, Vancouver, Canada; 2 Department of Pediatrics/Division of Neonatology, University of British Columbia, Vancouver, Canada; 3 Child & Family Research Institute, Vancouver, Canada; 4 Department of Pediatrics/Division of Neonatology, University of Toronto, Ontario, Canada; 5 Department of Pharmacy, Children’s & Women’s Health Centre of British Columbia, Vancouver, Canada; 6 Department of Pediatrics/Division of Cardiology, University of British Columbia, Vancouver, Canada; 7 Department of Statistics, University of British Columbia, Vancouver, Canada; 8 Department of Occupational Science and Occupational Therapy, University of British Columbia, Vancouver, Canada; Johns Hopkins Bloomberg School of Public Health, UNITED STATES

## Abstract

**Objective:**

To compare the effect of oral glucose given with or without facilitated tucking (FT), versus placebo (water) to facilitate image acquisition during a targeted neonatal echocardiography (TNE).

**Design:**

Factorial, double blind, randomized controlled trial.

**Setting:**

Tertiary neonatal intensive care unit (NICU).

**Patients:**

Infants born between 26 and 42 weeks of gestation (GA).

**Interventions:**

One of four treatment groups: oral water (placebo), oral glucose (25%), facilitated tucking with oral water or facilitated tucking with oral glucose, during a single, structured TNE. All infants received a soother.

**Main Outcome Measure:**

Change in Behavioral Indicators of Infant Pain (BIIP) scores.

**Results:**

104 preterm infants were randomized (mean ± SD GA: 33.4 ± 3.5 weeks). BIIP scores remained low during the echocardiography scan (median, [IQ range]: 0, [0 to 1]). There were no differences in the level of agitation of infants amongst the treatment groups, with estimated reductions in mean BIIP relative to control of 0.27 (95%CI -0.40 to 0.94) with use of oral glucose and .04 (-0.63 to 0.70) with facilitated tucking. There were also no differences between treatment groups in the quality and duration of the echocardiography scans.

**Conclusions:**

In stable infants in the NICU, a TNE can be performed with minimal disruption in a majority of cases, simply by providing a soother. The use of 25% glucose water in this context did not provide further benefit in reducing agitation and improving image acquisition.

**Clinical Trial Registration:**

Clinical Trials.gov: NCT01253889

## Introduction

Targeted neonatal echocardiography (TNE) is increasingly being use in the neonatal intensive care unit (NICU) to guide decision-making in the hemodynamic management of infants [1–3]. While this procedure may not be highly invasive, excessive agitation of infants during echocardiography may reduce image quality and prolong data acquisition, leading to misdiagnosis. In addition, infants admitted to the NICU are generally exposed to a high number of essential life-saving, yet stressful procedures. Poorly managed stress during the neonatal period can have major consequences on the developing brain [4–8]. Given the impact of cumulative stress at this critical age, experts recommend the use of effective stress reduction strategies during all routine procedures [9]. This may be even more important in infants born at earlier gestation, who show lower tactile thresholds, and in whom relatively less invasive procedures can induce significant stress responses [10–12].

Sweet solutions are the clinical standard for treating mild-to-moderate procedure-related stress in the NICU [9]. They are effective and have been recommended in both term as well as preterm infants [9, 13]. To our knowledge, no previous study has addressed the level of stress produced during a TNE. Anecdotally, many neonatologists will use oral glucose solutions in an attempt to settle the patient and improve the image acquisition process during a TNE, despite a lack of data supporting this practice [3]. Whether or not oral glucose provides tangible benefits in improving the performance of TNE has also never been demonstrated. Alternatively, facilitated tucking, a holding strategy whereby a nurse or caregiver provides gentle but firm containment of an infant's limbs, reduces standardized stress indices by 20–35% during a variety of routine NICU procedures and may provide greater benefits in the context of a TNE [14–17].

To address these knowledge gaps, we designed a randomized trial to determine the effectiveness of an oral administration of a 25% glucose solution given via a soother compared to placebo, with or without gentle facilitated tucking in reducing agitation in infants able to receive oral therapies, during a TNE. We hypothesized that a 25% oral glucose solution would reduce infant stress and agitation without increasing the duration or compromising the quality of echocardiography scan.

## Methods

### Population

The study (S1 Study Protocol) was approved by the Children’s & Women’s Health Centre of British Columbia (C&W) Research Ethics Board (#H10-02069). Informed consent was obtained (S1 Consent Form) from a parent/legal guardian of infants, in writing, in all subjects enrolled. Consent forms are kept on file and were also documented in a sequential log reporting all eligible participants anonymously, as standard and approved by our ethics committee. Infants were eligible if they were born between 26 and 42 weeks of gestation and admitted to the NICU (Level 2/3) or the Intermediate Nursery (Level 1/2a) at C&W. Infants were excluded if they had a lethal congenital anomaly, if they received analgesics or sedatives within 72 hours before randomization, if they had a history of maternal abuse of controlled drugs and substances, if they were unable to receive oral medications or if they were too unstable to have a TNE. Infants were enrolled between January 2011 and June 2013. Reporting of this trial follows the CONSORT statement (S1 CONSORT Checklist).

### Intervention

The study was a four-arm, double blind, factorial randomized controlled trial. Infants were allocated to one of four intervention groups: *Group C*: water solution only, *Group G*: oral glucose only, *Group FT*: facilitated tucking with water solution and *Group FT+G*: Facilitated tucking with oral glucose solution. Infants in all four groups also received a soother which was maintained in the infant’s mouth throughout the assessment. Randomization was done using an external computer-generated list of permuted sequential blocks of four. The intervention took place during a single, standardized TNE performed by experienced echocardiographers (PML, JT, MJ, EC and AS; structure of TNE provided as S1 Echocardiography Quality Scoring Sheet). Infants were allowed to rest for 10 minutes before handling. Then, two minutes before beginning the echocardiography, infants were given the study solution (0.5 or 1.0 mL for infants born at 26–31 weeks or 32–42 weeks of gestation, respectively). The study solution was applied to the anterior portion of the tongue followed by insertion of a soother. Facilitated tucking was done throughout the procedure by placing the infant's limb in a flexed, tucked position and providing containment through the use of the caregiver hands and a small blanket. During the study period, infants remained in their incubator/cot, positioned with rolls around the body to promote a flexed position. Administration of the study solution could be repeated during the procedure if the ultrasound operator felt that the scan could not proceed due to agitation, up to a maximum of four doses. To minimize disruption of the infant, sonographic gel envelopes were pre-warmed. No other interventions were provided unless the infant became unstable clinically. All infants were maintained on a standard cardiorespiratory monitor. To ensure the environmental influences were equivalent during the assessments, ratings of light, noise and general activity were performed using a modified version from the Newborn Individualized Developmental Care and Assessment Program [18]. Other than study pharmacist (EK) who generated the random allocation sequence and who was not involved in the intervention or data analysis, all other investigators and research staff remained blinded to the oral solution intervention during the trial and during the rating of outcomes. Both oral solutions were prepared in water and looked exactly the same. However, the facilitated tucking intervention could not blinded. The randomization and data management was carried out independently by data support staff at our research institute who had no role in the design of the trial or in the analysis of data.

### Outcome measures

The primary outcome was a change in Behavioral Indicators of Infant Pain (BIIP) scores across 4 phases of the echocardiography (*Baseline*, *Post-Solution*, *Mid-ECHO* and *After Recovery*). BIIP is a reliable, validated and widely used measure of stress in term and preterm infants, incorporating physiological as well as behavioral indices [19, 20]. BIIP scores range from 0–9, and is comprised of five precisely defined facial and two hand actions and behavioral states. We used a web-based sample size calculator (www.stat.ubc.ca/~rollin/ssize) which implements the customary large sample based calculations for a two group comparison of means. To ensure sufficient power to detect a small, minimal clinically significant difference of 2 points on the BIIP scale, we set a sample size of 25 infants per group, using a previously observed standard deviation of 2.4 in NICU infants [21] with 80% power (for a two-arm study) and 98% (for a four-arm study, allowing for co-variate adjustment if necessary) and alpha error of 5% to detect changes in BIIP score at Mid-ECHO. The trial ended when this sample size was reached, after completing an entire randomization block of eight.

Study phases were defined as follows: *Baseline*: 5 minutes before beginning of the echocardiography and before any physical contact with the infant, *Post-Solution*: two minutes after the first administration of the oral solution and just before the beginning of the echocardiography; *Mid-ECHO*: halfway through the echocardiography assessment (i.e. after completion of 4 and 5-chamber, and long-axis views), and *After Recovery*: immediately after completion of the echocardiography. BIIP scores were recorded by bedside videotaping and subsequently coded by trained research assistants. Video segments of both the procedure phases and the infants were randomized for viewing. Inter-rater reliability between 2 coders was assessed twice during the trial and was maintained at or above 0.85 (interclass correlation).

Secondary outcomes were changes in mean heart rate across procedure, number of doses of oral solution, total time to perform the echocardiography and quality of the images. Occurrence of cardiorespiratory events (e.g. apneas, desaturations) as well as any cardiovascular instability was also monitored for safety. For rating of the quality of the echocardiography scans, images were scored (maximum score of 62) by two experts neonatologists (PJM, AJ) not involved in data acquisition, and blind to treatment group and clinical characteristics of the infants. Images were rated according to a standardized grid that took into account whether specific view/axes were adequately imaged and whether the information obtained was interpretable, as well as whether the study quality provided was considered within the standard of care (satisfactory) or not (limited value or unsatisfactory; S1 Echocardiography quality scoring sheet). Averages of scores were used (inter class correlation 0.85).

### Statistical analyses

Baseline demographic and clinical information about the infants were compared using descriptive statistics. We used linear mixed effects models to perform three factor (glucose—yes/no; FT—yes/no; phase of echocardiography) repeated measures analysis of variance to assess overall effects on change from baseline in BIIP score and heart rate. Simple 2- way analysis of variance was used to derive 95% confidence intervals for treatment effects on change from baseline in BIIP score at the 3 post intervention times. In order to make an overall evaluation of the treatment effects over the post intervention phase, we originally performed a three way linear mixed effects analysis incorporating time (the repeated factor) and the two treatment factors taking the change from baseline measure as the outcome. As this analysis revealed no significant effects, we reverted to simple two-way analysis for the change score from baseline at each time point separately to provide confidence intervals. Also, we used a standard additive subject effect model resulting in a compound symmetry model. Reliability for BIIP and quality of the TNE images were analyzed by scores using interclass correlations. The analysis was done by intention-to-treat. Data were analyzed using R version 3.1.1 and R Studio Version 0.99.451 [22].

## Results

### Impact of intervention on primary outcome of stress

**Fig 1:**
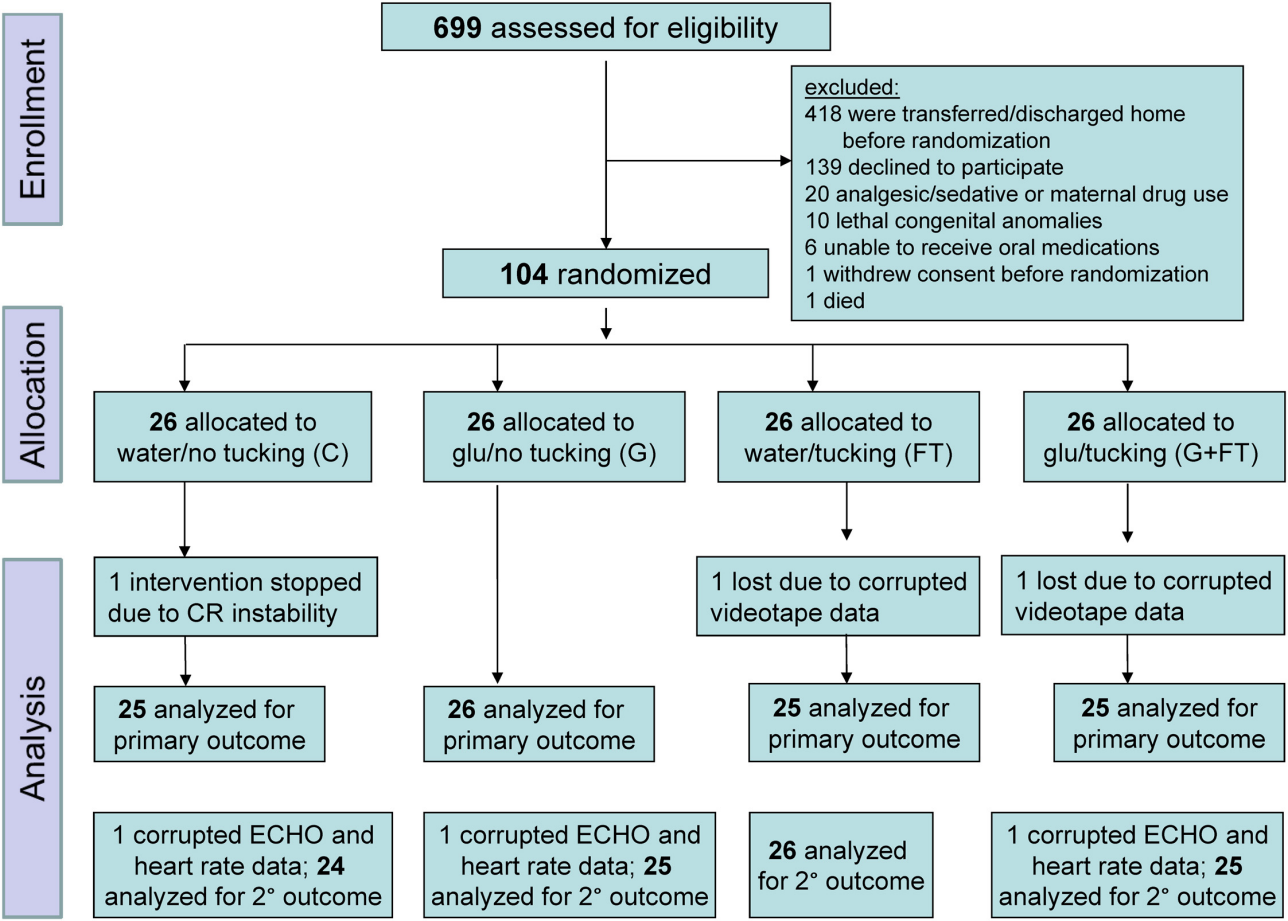
Study flow diagram.

The flow diagram for the study enrolment is presented in Fig 1. Of a total of 699 eligible infants, 418 were not enrolled because they were either transferred or discharged home before parents could be approached. A total of 38 were excluded, and of 243 infants approached for consent, 139 declined participation and the remaining 104 were randomized to one of four intervention groups. Of 104 infants randomized, the primary outcome could not be obtained in one infant in the water/no tucking group (unable to complete the echocardiography assessment due to cardiovascular instability—the infant recovered immediately after the study was stopped); and one infant in each of the water/tucking and glucose/tucking groups (due to technical problems with video recording). Therefore, 101 infants were included in the primary outcome analysis.

**Fig 2:**
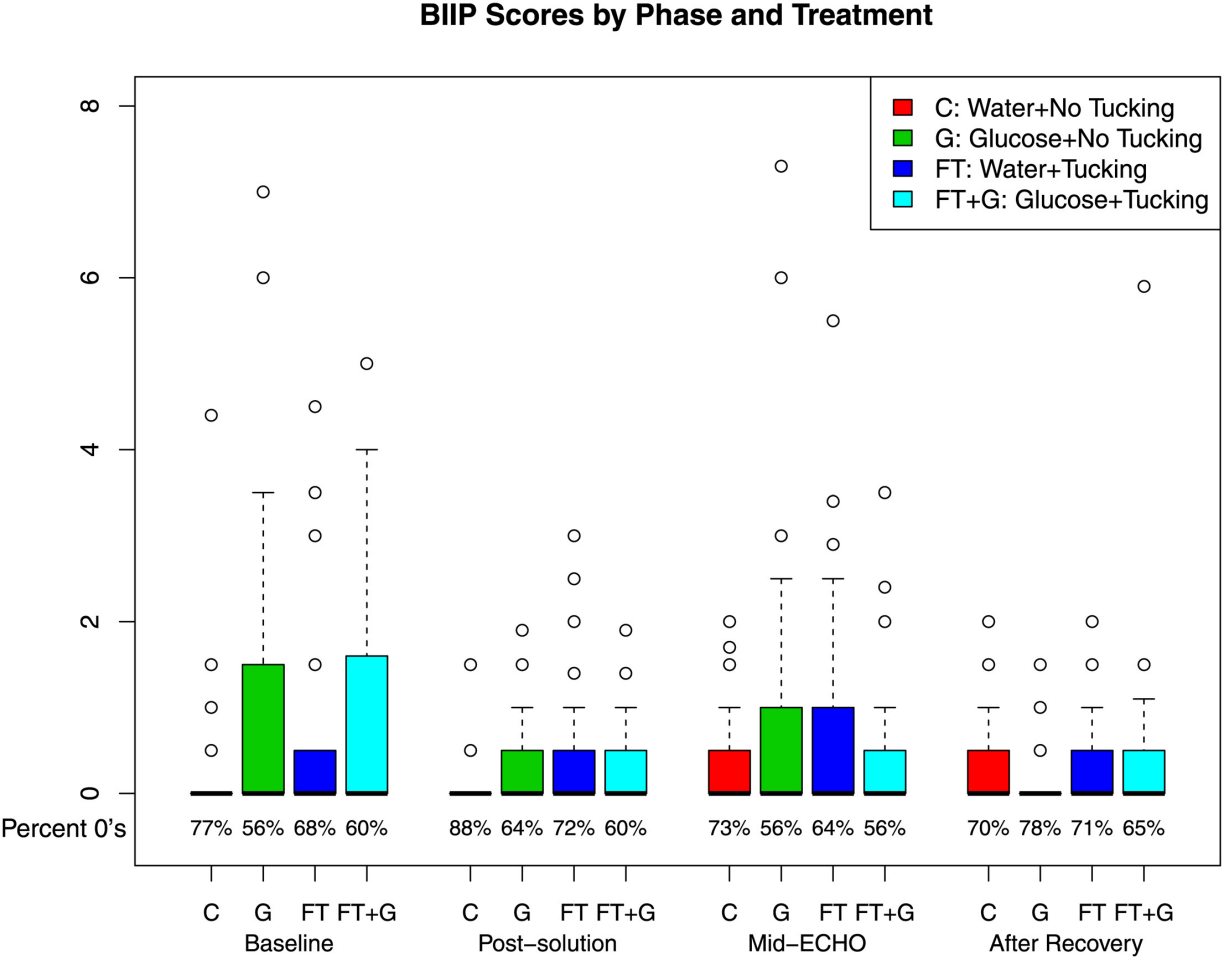
Boxplots for the Behavioral Indicators of Pain total scores by treatment group and echocardiography phase. Due to the preponderance of 0 values in the data, we also provide the percentage of such values below each boxplot.

Baseline characteristics of the infants randomized to each intervention group were comparable (Table 1). The environmental ratings (on a scale of 1–5) for the intensity of ambient light, the sound level and the level of surrounding activity were also similar between infants in all four intervention groups (Table 1). BIIP scores are shown in Fig 2 by treatment group and across intervention phases. Notably, BIIP scores remained low throughout the echocardiography, in all four treatment groups. There were no differences in the level of agitation of infants amongst the treatment groups, with estimated reductions in mean BIIP relative to control of 0.27 (95%CI -0.40 to 0.94) with use of oral glucose and .04 (95%CI -0.63 to 0.70) with facilitated tucking. Primary estimates for changes in BIIP across intervention phases were published previously, and also did not show vary significantly between groups [23]. There were no significant interaction effects between treatment and phase of echocardiography (not shown). We detected no additive effect of Glucose and FT on change in BIPP scores from baseline during the echocardiography (p>0.05).

**Table 1:** Clinical characteristics of infants randomized into study.

Characteristic		Water (n = 26)	Glucose (n = 26)	Water + FT (n = 26)	Glucose + FT (n = 26)
Gestational age, wks (mean ± SD)		33.3 ± 4.3	34.2 ± 3.6	32.9 ± 3.4	33.0 ±3.1
Birth weight, g (mean ± SD)		2070 ± 942	2202 ± 765	1869 ± 816	1892 ± 720
SNAP score at 24h (median [IQ range])		7 [0–12]	7 [0–14]	0 [0–10]	9 [0–12]
Day of age (median [IQ range])		15 [5–35]	10 [6–24]	14 [7–32]	10 [7–24]
Male sex, N (%, [95%CI])		18 (69, [48; 86])	15 (58, [37; 77])	14 (54, [33; 73])	20 (77, [56; 91])
Environmental rating(mean ± SD)	Light	3.2 ± 0.7	3.2 ± 0.9	3.2 ± 0.7	3.0 ± 0.9
	Sound	3.0 ±1.0	3.2 ± 0.7	3.3 ± 1.0	3.3 ± 0.8
	Activity	3.4 ± 1.0	3.5 ± 0.6	3.6 ± 0.8	3.4 ± 1.0
Respiratory support N (%, [95%CI])	None	22 (85, [65; 96])	24 (92, [75; 99])	20 (77, [56; 91])	24 (92, [75; 99])
	Nasal cannula	1 (3.8, [0; 20])	0	0	0
	C/Biphasic PAP	3 (12, [2.5; 30])	2 (7.7, [0.9; 25])	6 (2.3, [9.0; 44])	2 (7.7, [0.9; 25])
	Endotracheal ventilation	0	0	0	0

### Secondary outcomes

**Fig 3:**
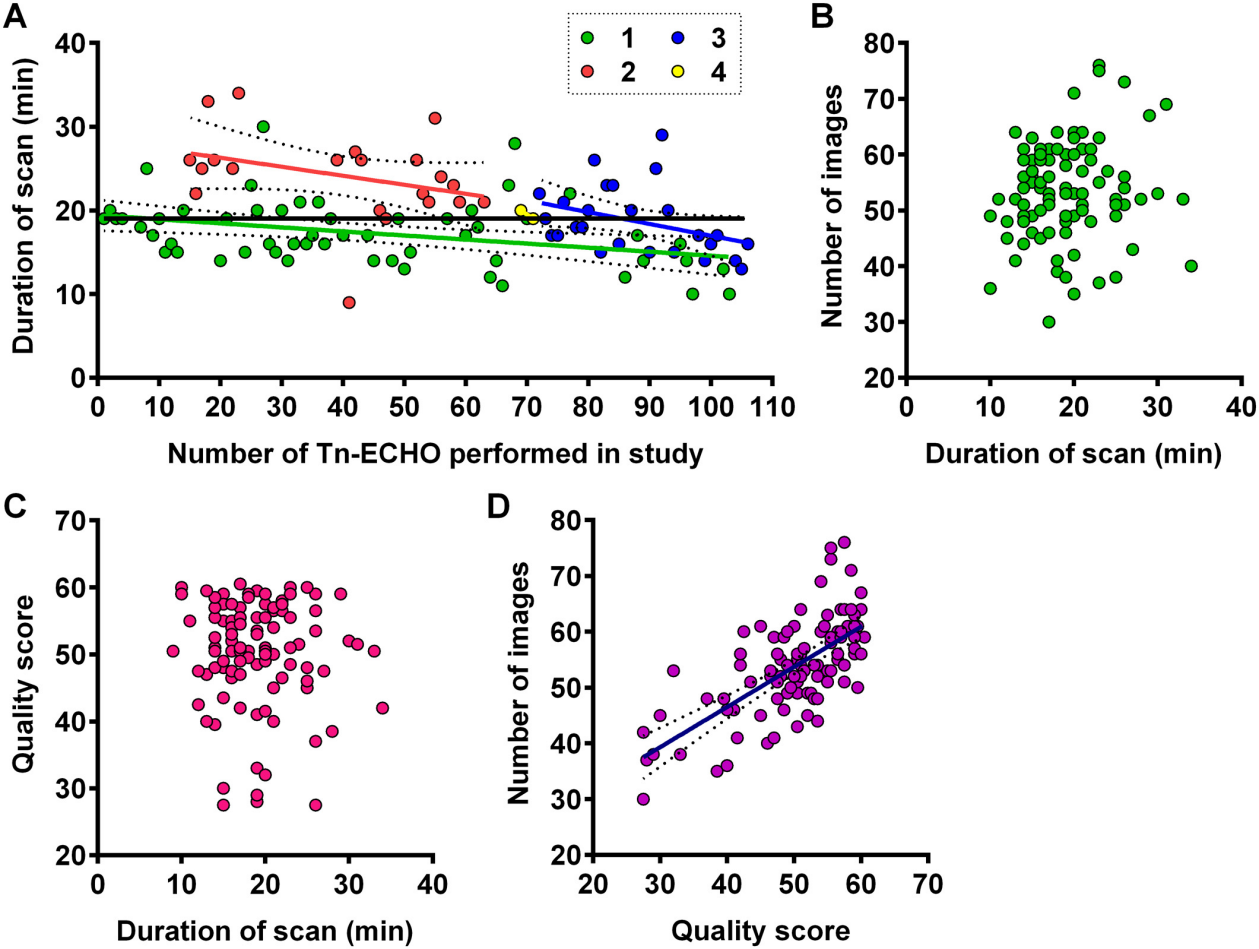
**(A)** Decrease in scan time among each TNE operator (1 to 4) with increasing number of Tn-ECHO scans over the progression of the study. Relationship **(B)** between quality and duration of Tn-ECHO scan, **(C)** between of number of images acquired and duration of Tn-ECHO scan, and **(D)** between quality of Tn-ECHO scan and number of images acquired. Linear regression (solid lines) with 95% confidence limits (dotted lines).

Four infants were excluded from the secondary outcome analyses due to echocardiography data corruption. Therefore, 100 infants were included in the secondary outcomes. Secondary outcomes are presented in Table 2. Overall, 47% and 70% of the echocardiography studies were judged “satisfactory” by each of two reviewers, respectively. We observed important differences in the duration of TNE comparing each operator (Fig 3A). There was no correlation between the duration of echocardiography study and the number of images acquired (Fig 3B), or the quality of the scans (Fig 3C). However, the number of images acquired was directly correlated with the quality of the echocardiography scan (Spearman r = 0.65; p<0.0001; Fig 3D).

**Table 2:** Secondary outcomes.

Outcome	Water (n = 24)	Glucose (n = 25)	Water + FT (n = 26)	Glucose + FT (n = 25)
Number of oral solution doses (mean ± SD)	1.2 ± 0.5	1.3 ± 0.5	1.3 ± 0.7	1.4 ± 0.6
Duration of echocardiography, min (mean ± SD)	19 ± 5.6	19 ± 4.4	20 ± 5.3	18 ± 4.7
Quality of echocardiography, score (mean ± SD)	49 ± 9	51 ± 9	51 ± 7	50 ± 8
Number of images (mean ± SD)	54 ± 10	52 ± 8	55 ± 8	54 ± 9
Change in heart rate*, BPM (mean ± SD)	-6 ± 14	-5 ± 14	-8 ± 14	-3 ± 14

*From baseline to end of echocardiography scan;

FT: Facilitated tucking.

When comparing groups, the number of doses of glucose versus placebo required during the echocardiography remained low in all four groups (Table 2). However, the quality and duration of the echocardiography studies remained comparable between treatment groups. Although there was a trend towards a greater number of satisfactory echocardiography studies in each of the two glucose treatment groups, the difference did not reach statistical significance (p>0.3). From baseline to end of echocardiography scan, the mean heart rate decreased in all four study groups. No effects of Glucose or FT were found at mid-ECHO comparing differences in BIIP between treatment versus control group (Glucose: 2.5 [CI: -3.7 to 8.6]; FT: -2.2 [CI: -8.3 to 3.9]) or post-recovery heart rate (Glucose: 2.0 [CI: -3.7 to 7.6]; FT: -3.3 [CI: -9.0 to 2.3]), in time to complete the echocardiography (Glucose: -1.0 [CI: -3.0 to 0.9]; FT: 0.6 [CI: -1.4 to 2.5]) or in the number of oral solutions given (Glucose: -0.06 [CI: -0.28 to 0.16]; FT: -0.10 [CI: -0.32 to 0.12]).

## Discussion

This randomized controlled trial is the first evaluating clinical interventions to improve the practice of TNE. Our study is also the first to systematically evaluate the level of stress experienced by infants during neonatal echocardiography. We reported the primary outcome of this trial in a short letter and demonstrate a lack of benefit of oral glucose on agitation of infants during a TNE [23]. Here, we present the entire trial details including the results of secondary outcomes. In contrast to a common perception, we demonstrate a lack of benefit of the use of oral glucose solution for improving the performance of an echocardiography. As an alternative, we show that it is feasible to use facilitate tucking while avoiding physical interference on the ultrasound operator and without compromising image quality or increasing the time required to complete the echocardiography assessment. In the context of family-centered care, parents could be encouraged to hold their child during an echocardiography, which may be beneficial both to the infant and the parent. BIIP is a highly sensitive measure of stress and agitation. Preventing all sources of acute and cumulative effects of stress is a high priority in neonatal intensive care [9]. The finding that stress levels are generally low in infants during a TNE is, in itself, of importance and of broad clinical relevance given the widespread and increasing use of TNE in the NICU [1, 3, 24]. The mechanism of action of oral sweeteners has been reported in animal studies, but remains less well understood in human infants in the context of stress [25, 26]. While the use of oral sweeteners is usually recommended for procedures of shorter duration (e.g. blood collection), research has shown their calming effects can last for up to an hour even in more mature infants [27].

Other studies have addressed the physiological stability of infants during a TNE and provided data supporting the safety of this procedure even in the smallest infants [28]. On the other hand, concerns have been expressed that repeated use of oral sweeteners may have adverse effects in preterm infants [27, 29, 30]. Indeed, we found that infants in the glucose group showed a trend towards an elevated heart rate although the difference was not statistically significant (Table 2). This finding does indeed suggest a pharmacological response to our intervention, and is consistent with a recent trial conducted in preterm infants where a single dose of sucrose given to manage pain was shown to increase, rather than decrease heart rate and markers of oxidative stress [31].

Our study has some limitations. Infants included were relatively stable as none of the infants were on mechanical ventilation. Moreover, infants who were too immature or too unstable to be able to safely ingest oral glucose were excluded. Indeed, in our NICU, like many other NICUs across North America, administration of oral medications to ventilated infants or in infants on CPAP is outside the standard of practice. One out of 104 infants did not tolerate the procedure or intervention because of cardio respiratory instability. However, the infant recovered quickly after the intervention was discontinued and it is not entirely clear whether the instability was due to the echocardiography procedure, the administration of the oral solution, or another unrelated event. While the level of stress during an echocardiography procedure may differ in a critically ill ventilated infant, other stress-reducing measures could be considered and the risks/benefits of any procedure should be carefully assessed. For other more vigorous infants in whom clinicians often consider the use of oral glucose, findings of our study are directly applicable to the vast majority who can receive oral solutions.

In conclusion, TNE can be performed in stable infants in the NICU with minimal disruption simply by providing a soother for comfort. The use of 25% glucose water in this context did not provide further benefit in reducing agitation and improving image acquisition. Similarly, facilitated tucking appeared safe and feasible without demonstrated additional benefit in reducing echocardiography-related stress in the infant.

## Supporting Information

S1 Consent Form (PDF)

S1 CONSORT Checklist (PDF)

S1 Echocardiography Quality Scoring Sheet (PDF)

S1 Study Protocol (PDF)

## Abbreviations

TNE: Targeted Neonatal Echocardiography
NICU: Neonatal Intensive Care Unit
BIIP: Behavioral Indicators of Infant Pain
FT: Facilitated tucking
