# Supplementary material for: A Randomized Controlled Trial of the Use of Oral Glucose with or without Gentle Facilitated Tucking of Infants during Neonatal Echocardiography
Source: PLoS One. 2015 Oct 23;10(10):e0141015. doi: 10.1371/journal.pone.0141015 (PMC4619855; doi:10.1371/journal.pone.0141015)
Supplement: S1 Consent Form — (PDF) [file pone.0141015.s001.pdf]

INFORMED CONSENT

Project Name: Sweetheart Study

Principal Investigator: Dr. Liisa Holsti, Dept of Occupational Science and Occupational Therapy, UBC. (604) 875-2000, local 5200
Co-Investigators: Dr. Pascal Lavoie, Dept. of Pediatrics, University of British Columbia
Dr. Amelie Stritzke, Clinical Fellow NICU
Dr. Derek Human, Dept. of Pediatrics, University of British Columbia
Dr. JP Collet, Dept. of Pediatrics, University of British Columbia

Study Coordinators: Linda Dix-Cooper, NICU Research Coordinator
Eddie Kwan, Clinical Pharmacist
Alice van Zanten, NICU Research Nurse

Introduction

You have been invited to participate in this study because your baby is being cared for in the intensive care or intermediate care nursery. Cardiac ultrasound is a non-invasive medical test which tells doctors how well a baby's heart is working. Sometimes sick babies in the nursery need a cardiac ultrasound. This test may cause some stress because of the handling (movement of a baby by health professional staff) needed to do the test. Furthermore, an infant's own movements during the test could prolong the time needed to finish and can even reduce the quality of the test.

To calm babies, reduce stress and to shorten the ultrasound time, some doctors regularly use small amounts of sugar and water (glucose) and gentle holding (facilitated tucking). Although we know that sugar water given by mouth and facilitated tucking work to calm babies generally, we do not know if they work during a cardiac ultrasound. Because we don't know for certain, glucose is not given routinely by all doctors for this purpose. This may result in unnecessary stress in babies. Also, we do not know if it really helps doctors complete the cardiac ultrasound faster or more accurately.

In this study, our goal is to determine whether sugar water given by mouth and facilitated tucking work in calming babies during a cardiac ultrasound.

Your participation is entirely voluntary. If you decide not to participate in this study you do not have to provide reasons and your decision will in no way affect the quality of care your baby is entitled to receive. Also, you may withdraw from the study at any time if you decide otherwise.

Study Procedures

Throughout the procedure, your baby's face and arms will be videotaped. Then, just before the treatments are given, without disturbing him/her, a nurse will assess your baby's behavioral stress levels using a standard test. After this assessment, your baby will receive one of four treatments: 1) sugar water with a soother on its own, 2) sugar water, soother and facilitated tucking or 3) a placebo (i.e. just plain water) by mouth and soother on their own or 4) plain water, soother and facilitated

tucking, before the start of the cardiac ultrasound. Doctors can give more glucose or water up to four more times during the ultrasound if your baby appears to need help staying calm.

Sugar water or plain water will be given 0.5ml (equivalent to 1/10th of a teaspoon) at a time for babies 26-31 weeks gestation or 1.0ml (equivalent to 1/5th of a teaspoon)for babies 32-42 weeks gestation. The solution will be given slowly into your baby's mouth and then he/she will be given a soother to suck on throughout the ultrasound. During the testing (estimated about 20-30 min), we can give your baby a total of less than ½ a tsp if he/she was born between 26-31 weeks, and less than 1 tsp if he/she was born 32-42 weeks of age.

If your baby receives facilitated tucking, the research nurse will gently hold your baby's arms and legs close to their body to help keep your baby calm.

If you choose not to participate in this study, your baby will get a soother, but may or may not receive sugar water if agitated during the cardiac ultrasound and other calming measures don't work.

After the bedside procedure is over, your baby's videotape will be analyzed by research staff who will not know the identity and the medical history of your baby. As well, we will provide you with a de-identified non-diagnostic image of your infant's heart to keep.

Risks
There is a very small risk that your infant might have changes in their heart rate or breathing when given the sugar or plain water (< 1%). However, your baby will remain on standard nursery heart rate and breathing monitors throughout the testing. If he/she shows any difficulties, the testing will be stopped. There are no other known side effects of feeding sugar water or water to premature or term infants. There are no known side-effects from the cardiac ultrasound.

There is an extremely small risk of choking (less than 1%) during the administration of the soother or sugar water/placebo. If a serious choking spell occurred, your baby would be immediately attended to by a nurse and the study would be stopped.

Possible Benefits
Participation in this study may not be of any direct benefit to you or your infant; however, receiving sugar water may reduce your infant's stress. Your participation should add to our current understanding of the ways of reducing stress responses in infants cared for in the neonatal intensive care nursery. We will provide you with a non-diagnostic image of your infant's heart on a USB stick.

Confidentiality
You/Your child's confidentiality will be respected. Only members of the research team will have access to your baby's videotapes. No personal information about your baby will be indicated on the videotapes; each baby will be given a unique study number to protect his/her identity. The videotapes will be kept in Dr. Holsti's office in a locked cabinet. The videotapes will be kept for 5 years after the study and then they will be erased. There are no plans for uses of the videotaped data other than those listed in this study. No information that discloses you/your child's identity will be released or published without your specific consent to the disclosure. However, research records and medical records identifying you/your child may be inspected in the presence of the Principal Investigator or her

designate by representatives of Health Canada, and the UBC Research Ethics Boards for the purpose of monitoring the research. However, no records which identify you/your child by name or initials will be allowed to leave the Investigators' offices. Any information resulting from this research study will be kept confidential and will be retained in a secure information system and locked filing cabinet. Only research staff will have access to the study data.

Remuneration or Reimbursement
Your participation in this study is voluntary, participation in this study will not cost you anything, nor will you be offered any payment for your participation.
In signing this document you are in no way waiving my or my child’s legal rights against the sponsors, investigators, or anyone else.

Contacts
If you have any questions about the research, please contact Dr. Liisa Holsti, Principal Investigator Tel: (604) 875-2000 local 5200 or Alice van Zanten, Research RN Tel:604-875-2000 local 6909/ Pager: 41-01488. Any concerns about your/your baby’s treatment as a research subject can be referred to the Research Subject Information Line, Office of Research Services, UBC, toll-free Tel:1-877-822-8598 or by email at RSIL@ors.ubc.ca.

Consent

I have read and understood this informed consent form. I have had the opportunity to ask questions and to discuss this study with the research team, and my questions have been answered to my satisfaction. I understand that my participation in the above study is entirely voluntary, and that I may refuse to participate, or may withdraw from the study at any time without any consequences to my involvement with B.C.'s Children's & Women's Hospital. If at anytime you choose to withdraw from the study, the videotape of your infant during the study will be destroyed. I have been told that I will receive a signed and dated copy of this consent form for my own records.

I consent to participation in this study and to the review of my baby's medical records for the purposes of this research, and in signing this document I am in no way waiving my or my child's legal rights against the sponsors, investigators, or anyone else.

Name of infant: ______________________________________

____

Parent's signature Printed name Date

____

Signature of Person Obtaining Consent Printed name Date

____

Signature of Principal Investigator/Delegate Printed Name Date
