# Supplementary material for: A Randomized Controlled Trial of the Use of Oral Glucose with or without Gentle Facilitated Tucking of Infants during Neonatal Echocardiography
Source: PLoS One. 2015 Oct 23;10(10):e0141015. doi: 10.1371/journal.pone.0141015 (PMC4619855; doi:10.1371/journal.pone.0141015)
Supplement: S1 Echocardiography Quality Scoring Sheet — (PDF) [file pone.0141015.s003.pdf]

Sweetheart Study ECHO Scan Score Sheet

Study ID: ______________

Date: ______________ Assessor: ____________________

(2: ADEQUATE, 1: LIMITED IMAGE OR INCOMPLETE, 0: MISSING)

4-Chamber view:

- 2-D 4-chamber view of sufficient quality for assessment of:
  - Atrial and ventricular sizes (both left and right ventricles) -------- (2, 1, 0)
  - Left and right ventricular contractility ------------------------------ (2, 1, 0)
  - Tricuspid and mitral valves -- (2, 1, 0)
  - Large A-V SD --- (2, 1, 0)
- Color Doppler assessment of tricuspid flow ------------------------------- (2, 1, 0)
- Interrogation of tricuspid valve flow using pulse/continuous Doppler if significant stenosis/regurgitation (as appropriate) ------------------------ (2, 1, 0)
- Color Doppler assessment of mitral flow ---------------------------------- (2, 1, 0)
- Interrogation of mitral valve flow pattern using pulse/continuous Doppler (whichever appropriate) -- (2, 1, 0)

5-Chamber view

- Color Doppler assessment of aortic flow ---------------------------------- (2, 1, 0)
- Pulse/continuous Doppler measurement of left outflow tract ------------ (2, 1, 0)
  (adequate angle?) --- (Yes, No)

Left parasternal long-axis view:

- 2-D image of sufficient quality for assessment of:
  - Ventricular sizes and contractility (including Fract. Shortening) - (2, 1, 0)
  - Left atrial size and estimation of LA/Ao ratio using M-mode ----- (2, 1, 0)
  - Measurement of aortic valve diameter (using 2D or M-mode) ---- (2, 1, 0)
  - Determination of relative position of great vessels ----------------- (2, 1, 0)
- Color Doppler of ventricular septum (defect) ----------------------------- (Yes, No)

- Long-axis tricuspid valve with color Doppler (and use of pulse/continuous Doppler whenever appropriate) ---------------------------- (2, 1, 0)
- 2-D view of pulmonary outflow tract, and color Doppler to measure velocity whenever appropriate -- (2, 1, 0)
- M-mode LA/Ao ratio -- (2, 1, 0)

Short axis view:

- 2-D images of sufficient quality for assessment of:
    - Aortic valve -- (2, 1, 0)
    - Left/right ventricular shape, septal curve and contractility -------- (2, 1, 0)
    - Pulmonary bifurcation -- (2, 1, 0)
- Measurement of pulmonary flow using color Doppler --------------------- (2, 1, 0)
- Measurement of pulmonary flow by continous/pulse Doppler (as appropriate) -- (2, 1, 0)
- M-mode across left ventricle in short-axis (to calculate fract. Shortn.) -- (2, 1, 0)
- Pulmonary veins with color Doppler (short- OR long-axis views) ------ (Yes, No)

High parasternal arch/ductal view:

2-D image of sufficient quality for assessment of:

- Main/branch pulmonary arteries -- (2, 1, 0)
- Patent ductus arteriosus (exclusion of a PDA) ------------------------ (2, 1, 0)
- Aortic arch caliber and dimension -- (2, 1, 0)

- 2-D assessment of ductal diameter *(score 2: if not indicated)* ------------ (2, 1)
- Assessment of ductal flow by color Doppler *(same)* ----------------------- (2, 1)
- Transductal flow and direction (pulse Doppler) *(same)* ------------------- (2, 1)
- Interrogation of post-ductal aortic flow using color/pulse Doppler ------ (2, 1, 0)
- 2-D view of superior vena cava -- (Yes, No)

Sub-xyphoid view:

2-D image of sufficient quality for assessment of:

- Atrial septum/shunt (including color Doppler when appropriate) - (2, 1, 0)

- Doppler interrogation of SVC flow --- (Yes, No)

Last modified: July 2, 2013

Total score _____/62

Optimal Image Acquisition?

- Selection of correct probe and frequency ------------------------------------ (Yes, No)
- Use of gain ok (not too high – bright images, acceptable, too low dark images) ------------------------------------ (Yes, No)
- Depth penetration ok (does the image adequately fill the screen, too little or too much zoom) ------------------------------------ (Yes, No)
- Ordered sequence of imaging (they need to have an order – always in correct order, sometimes, no order) ------------------------------------ (Yes, No)
- Appropriate use of videoclips (2D images) vs still images (M-mode, Doppler) ------------------------------------ (Yes, No)
- Use of PWD and CWD as appropriate ------------------------------------ (Yes, No)

Overall, is this study is S: satisfactory, L: limited or N: not interpretable? ______

Specific comments: ____

____

____
