# Supplementary material for: A Randomized Controlled Trial of the Use of Oral Glucose with or without Gentle Facilitated Tucking of Infants during Neonatal Echocardiography
Source: PLoS One. 2015 Oct 23;10(10):e0141015. doi: 10.1371/journal.pone.0141015 (PMC4619855; doi:10.1371/journal.pone.0141015)
Supplement: S1 Study Protocol — (PDF) [file pone.0141015.s004.pdf]

PROTOCOL

**Project Name:**	Glucose 25% and facilitated tucking for reducing procedural stress during neonatologist-performed cardiac echocardiography in infants in the NICU
**Project Nickname:**	Sweetheart Study
**Principal Investigator**:	Dr. Liisa Holsti, PhD OT (R), Developmental Neurosciences and Child Health, Child and Family Research Institute and Dept. of Occupational Science and Occupational Therapy, UBC<br>Phone: 604-875-2000 ext 5200<br>Fax: 604-875-2384
**Co-Principal Investigator**:	Dr. Pascal Lavoie, MD PhD FRCPC<br>Associate Professor in Pediatrics,<br>Neonatologist Children's & Women's Health Centre of BC<br>Clinician-Scientist, Child & Family Research Institute
**Study Coordinator:**	Jennifer Claydon, NICU Research Coordinator<br>Alice van Zanten, NICU Research RN<br>Eddie Kwan, Clinical Pharmacist
**Expected Timeline**:	Nov 2010 to March 2014

Background

In order to ensure their survival, preterm infants need to receive intensive care and therefore necessarily undergo repeated episodes of stress from therapeutic procedures. Converging evidence from animal and preterm infant studies suggests that chronic exposure to stress may alter brain development and may contribute directly and indirectly to the developmental impairments found later in this population.[1,2]

For preterm infants, severe pain, such as post-operative pain, is controlled with pharmacological agents,[3,4] while stress associated with minor procedures, such as suctioning or heel lance, is managed with non-pharmacological interventions such as sweet solutions given orally (e.g. glucose or sucrose) and facilitated tucking.[5,6,7,8,9,10]These interventions need to be given prophylactically in order to be effective, have few associated adverse effects and are readily available. Importantly, new research indicates that sweetening agents may be most effective acting as sedatives (rather than analgesics), which makes them useful for comforting infants during stressful procedures which do not break the skin, especially when other modalities such as full maternal holding, etc. cannot be employed (i.e. during an ultrasound procedure).[11]

The practice of targeted neonatal echocardiography (tn-ECHO) is rising in the intensive care unit for rapid, serial cardiac bedside assessment of cardiovascular function and response to therapy in the NICU and as an adjunct to evaluations normally performed by a Pediatric Cardiologist. Neonatologists use tn-ECHO to gain information about cardiac function, pulmonary pressures, and in common neonatal conditions such as persistent ductus arteriosus, as an extension of their clinical exams to improve clinical decision-making.

A standard tn-ECHO can take up to ½ hour to complete depending on compliance of the infant. In several NICUs across Europe and the USA, it has become common practice to soothe the infants with non-pharmacological interventions and, at times, with glucose water and facilitated tucking, *although evidence directly supporting this practice is absent, and the effectiveness of these types of interventions has not been formally studied.* Importantly, these interventions are thought to be useful not only to comfort the infant, but also to minimize infants' movement which may significantly decrease the amount of time required to complete the tn-ECHO. Increased infant movements during the procedure may even alter the hemodynamic profile of the heart complicating interpretation of the data.

This study aims to exploit this potential sedative effect of glucose and calming effects of facilitated tucking for managing minor procedural stress. Here we will determine whether the use of 25% glucose water with or without facilitated tucking reduces stress and performance time during tn-ECHOs in infants in the NICU.

Purpose & Objective

<u>Primary Objective:</u> To compare the effect of a 25% glucose solution given via a soother with or without facilitated tucking with a similarly administered water placebo (control condition) on infant stress responses during and immediately after a tn-ECHO.

Secondary Objectives: To compare the performance time of a standard-defined tn-ECHO, the total number of times solutions are given, and the overall quality of the tn-ECHOs between treatment groups.

Hypotheses

1. When given 25% glucose, soother and/or facilitated tucking during functional echocardiography, infants will show the lower behavioral stress scores than infants in the control groups.

2. When given 25% glucose, soother and/or facilitated tucking during functional echocardiography, the time taken by neonatologists to conduct the procedure will be less than for infants in the control groups.

3. The number of repeated application of the solution will be lower in the infants receiving the glucose solution.

4. The quality ratings of the tn-ECHOS will be higher in the group of infants who receive sucrose+soother/and or facilitated tucking).

Study Design

Fatorial 4-arm, randomized double blind controlled study.

Inclusion Criteria

Infants born between 26-42 weeks of gestational age admitted to the Neonatal Intensive Care Unit and Intermediate Nursery at Children's and Women's Health Centre of British Columbia (C&W) who are stable enough to have a tn-ECHO. To avoid bias in completing time of the tn-ECHO we have excluded purely educational studies.

Exclusion Criteria

Infants who have congenital anomalies, or a lethal condition in whom intensive care is not indicated; infants who have received analgesics or sedatives within 72 hours of the assessment; history of maternal abuse of controlled drugs and substances. Infants who are too unstable to be exposed to a tn-ECHO or who already have an ECHO performed by a Pediatric Cardiologist within 4 hours. Inability to obtain parental consent (due to language barrier, etc.).

Methods

Parents will be asked for written informed consent prior to randomization. It is not standard of practice to obtain parental consent for procedures like echocardiography that are clinically indicated; therefore, the consent will be purely for research purposes.

One hundred and four infants will be randomized to one of four intervention groups (Grp 1: glucose + soother; Grp 2 glucose + soother + facilitated tucking; Grp 3: water + soother; Grp 4: water + soother+facilitated tucking) which will take place during a single tn-ECHO examination, at the discression and after consultation with the medical team.

Randomization to each group will be determined by generating randomly permuted sequential blocks of four and six allocation numbers. The Clinical Services Research Unit will initiate the randomization process and provide the information regarding group allocation that will guide the intervention. Access to the randomization list will be through a web-based system that will enable online interactive checking of the inclusion/exclusion criteria before authorizing random allocation. This system is very reliable with 24-hour on-line support; as part of the data management services, a person will be available by phone during working hours at C&W to provide back-up for the system for unanticipated events. Randomization and preparation of the solutions will then be done by the pharmacist on site who will provide the solutions in unmarked syringes containing 5ml of either water or 25% glucose.

Procedures

All testing will take place in the NICU and Intermediate Nursery at C&W. As in our previous studies, five minutes before, during and five minutes after the tn-ECHO procedure, a close up image of each infant's face and upper extremities are videotaped. The digital, color camera is set up using a tripod. Using a foot pedal attached to the camera, recordings are synchronized to mark events. These tapes will be used to provide blinded behavioral coding of our primary outcome measure in conjunction with our bedside scoring described below.

For all groups, each infant will remain in his/her incubator/cot, positioned with rolls around the body to promote a flexed position. Every attempt will be made to have the procedure scheduled one hour post feed, if the infant is feeding. After a 20 minute rest period, five minutes before the contact of the physician to conduct the examination, an attending physician who is not conducting the tn-ECHO will assess the infant's baseline stress levels over a 30 second period using the Behavioral Indicators of Infant Pain (BIIP).[12,13,14.] BIIP score will then be measured 2 min after first administration of the solution (at time of starting of the tn-ECHO), then halfway through the assessment (as defined per echocardiographical images obtained, after completion of the right ventricular long axis view with RVO), and finally immediately after the last contact of the physician.

With respect to the glucose/water administration, two minutes before the first contact by the physician performing the tn-ECHO, each infant will be given either 0.5ml of 25% glucose (26-31 weeks gestational age group) or 1.0 ml of 25% glucose (32-42 weeks gestational age group). The solution will be applied to the anterior portion of the tongue followed by insertion of a soother. The soother will be held in the infant's mouth to ensure that continuous contact is maintained throughout the testing period. During the course of the assessment (estimated about 20 min), this application can be repeated if necessary (if the infant's movements interfere with the quality of picture obtained) to a maximum of four times, adding up to max 2ml of 25% glucose in the 27-31 weeks gestational age group or 4ml of 25% glucose in the 32-40 weeks gestational age group. During each tn-ECHO, infants have additional handling only if it is required to maintain physiological stability. Pharmaceutical interventions are an exclusion criterion for the study.

For the infants randomized to receive facilitated tucking throughout the procedure, the bedside nurse will provide gentle, firm containment of the extremities. The facilitated tucking will commence immediately after the baseline BIIP score is taken, but before the glucose/water is administered. The NICU nurses have all been trained to use this technique for other procedures and so are very familiar with the technique. The nurse will continue to hold the infants until the tn-ECHO is complete.

Scans will be done by neonatologists or neonatologists in training (fellows) trained in tn-Echo. It will always comprise the following standardized assessment:

- Left ventricular performance (Contraction in 4- and 5-chamber views, long and short axis view, VTI, LVO, fractional shortening, IVRT, E:A ratio)
- Right ventricular performance (Contraction 4- and 5-chamber views, long and short axis view with RVO, septum deviation)
- Shunts (PDA: Presence, flow pattern, size and peak velocity; PFO/ASD/VSD)

End-organ blood flow: Celiac artery Doppler, MCA Doppler

Following each procedure, infants will be monitored for a 5 minute recovery period. One minute following the examination, the infant's stress will be measured again using the same standardized scoring system (BIIP; see below).

Data will be collected using a data collection sheet. Timing of tn-ECHO will be recorded as initiation of echo defined as application of the probe to the infant's chest. End time of echo will be recorded when the probe is removed from infant's chest. Time and amount of solution as well as application of soother and facilitated tucking, BIIP scores and adverse events will be also recorded on the data collection sheet. Upon completion of study, the research nurse will complete an environmental score, documenting the maximum environmental noise, to provide information regarding outside factors that could influence the infants' stress level during the procedure (i.e. light, noise, etc.). If desired by the family, we will provide them with a de-identified non-diagnostic image of their infant's heart to keep.

Outcome Measures

Primary Outcome Measure

The primary outcome measure will include changes in BIIP scores across phases (Baseline, maximal score at first contact by physician, ½ through the tn-ECHO, Recovery) of the study. The Behavioral Indicators of Infant Pain (BIIP) is a reliable and valid scale we developed for assessing acute procedural pain in preterm infants, [13,14]although it can also be used for measuring stress.

The BIIP combines into a single scale changes in sleep/wake states, five facial actions and two hand actions. The scoring sheet is appended. Scores range between 0-9, with scores 0-2 indicating mild or no pain/stress, 3-6 indicating moderate pain/stress and scores 7-9 indicating more severe pain/stress. In response to heel lance, under conditions where no intervention is provided, mean scores on the BIIP were 5.3 ± 2.6 points. For less intrusive, but stressful procedures, such as diaper change, mean BIIP scores were 3.1 ± 0.5. To ensure reliability for

BIIP coding, physicians will be trained by Dr. Holsti to achieve inter-rater reliability on the scale to above 0.85 (kappa). After the first 20 sessions, inter-rater reliability will be checked using the Bland and Altman approach which analyzes the differences in scores between two coders over the pair-wise mean of the two coders.[15]

From the bedside videotapes, using the Noldus Observer system[16,] one, 30-second block will be coded in real-time across four procedure phases:

Baseline	[T0] = 30 seconds, 5 minutes before first contact by the physician,
Time 1	[T1] = 30 seconds beginning 2 minutes after first solution given,
Time 2	[T2] = 30 seconds after 4 and 5 chamber views finished
Recovery 2	[T3] = 30 seconds after the last contact by the physician

As we have done in other studies, study phases will be randomized for viewing. Two graduate student video coders will be trained to achieve inter-rater reliability on the BIIP to above 0.85 (kappa) by our research nurse who has extensive experience scoring theBIIP.To ensure inter-rater reliability is maintained, during the trial, an additional 20% of the video segments selected randomly will be scored by the research nurse and the video coders. Inter-rater reliability will be assessed using the intra-class correlation (ICC), and graphically using the Bland and Altman approach by plotting the pair wise differences in scores between two coders against subject specific means over the two coders [15].

Secondary Outcome Measures
The secondary outcome measures will be the time to complete a standard tn-ECHO assessment, which will be defined when ultrasound images have been acquired for all four cardiac views (see appendix), the total number of times solutions are given to the infants, and the quality of images.

<u>Quality of tn-ECHO images:</u> The quality of tn-ECHO images will be rated by 2 neonatologists/cardiologists blind to the treatment groups and to any clinical information about each patient. A scale for rating the overall quality was designed (attached) in consultation with the Cardiology Department. Before the start of the study, a pilot of 10 tn-ECHOs will be rated to ensure starting inter-rater reliability is achieved (agreement > 85%). Then, inter-rater reliability will be checked between raters after ½ the study patients are enrolled. Inter-rater reliability will be assessed using the Bland and Altman approach which analyzes the differences in scores between two coders over the pair-wise mean of the two coders.

Other Data Collected

We will collect clinical information regarding the infant from birth to day of testing including but not limited to the following: birth weight, GA at birth, Apgar score at one minute, illness severity, head scans/MRI results, respiratory support, medications, type and time of last handling just before tn-ECHO. Much of this data will be used for descriptive purposes.

Sample Size Estimate

In infant studies where self-report is not possible, a 2 point difference in pain scores has been considered clinically significant (10% reduction).[17] Twenty five infants per group (100 total) will yield 80% power for a difference corresponding to the anticipated difference of 2 points on the

BIIP scale between treatment groups. Allowing for potential attrition of 10% and the need for including one or two co-variates in the analysis (e.g. differences in behavioral state at baseline), we may need to recruit up to 115 infants.

Statistical Analyses

Demographic and medical information about the infants will be compared between groups to ensure balance. Then, the primary outcome for analysis will be defined as the between group difference between average BIIP scores in the pre and during echocardiography observations. Mean change scores will be calculated and a 2 Group (Intervention) by Phase (Baseline, Echo) analysis of variance will be used to determine main effects. The confidence interval for the difference in treatment effects will be of primary interest. Any differences in baseline BIIP scores ormedical/demographic variables found would be included as co-variates in the analysis. Differences between tn-ECHO times between groups, in the total number of times solutions are administered for each group, and in the overall quality ratings of the tn-ECHOs will be compared using paired t tests.

Risk Attributed to the Study

The practice of tn-ECHOs for clinical purposes, as well as the administration of glucose water is considered standard of practice in many NICUs around the world (personal communications with neonatologists routinely performing tn-ECHOs at the Sick Children Hospital in Toronto, France, California US), and therefore, our study represent no greater risk (i.e. minimal risk study) to infants. Nevertheless, an extremely low risk of physiological instability (heart rate < 100 beats/minute or > 190 beats/minute, oxygen saturations below 85, regurgitation, choking or cyanotic spells) can occur when giving sweet solutions to preterm infants (<1%). [18]All infants will be in the presence of a physician and will remain on bedside monitors throughout the study; we will document any serious incidences of cardiovascular instability during the study. Should serious cardiovascular instability occur, the study intervention will be stopped.

Significance/Impact

Chronic stress as a result of undergoing repeated procedure related stress in infants cared for in the NICU is linked to adverse developmental outcomes. Our aim is to provide a simple method of calming, capitalizing on the potential soothing properties of glucose given in small doses, and facilitated tucking, and to generate evidence support the widespread use of these interventions during tn-ECHOs internationally. Moreover, infants who are more stable during cardiac functional echocardiography move less which may also reduce the time of assessment, thereby reducing the resource allocation of the physician and ensuring higher quality readings.

References

1. Bhutta AT, Anand KJS. Vulnerability of the developing brain. Neuronal mechanisms. *ClinPerinatol* 2002;29:357-372.

2. Peterson BS, Anderson AW, Ehrencranz R, et al. Regional brain volume abnormalities and their later neurodevelopmental correlates in term and preterm infants. *Pediatrics* 2003;111:939-948.

3. Scott CS, Riggs KW, Ling EW, et al. Morphine pharmacokinectics and pain assessment in premature babies. *J Pediatr* 1999;135(4):423-429.

4. Stevens B, Gibbins S, Franck LS. Treatment of pain in the neonatal intensive care unit. *Pediatr Clin North Am* 2000;47(3): 633- 650.

5. Stevens B, Yamada J, Ohlsson A. Sucrose for analgesia in newborn infants undergoing painful procedures (Cochrane review). *Cochrane Database Syst Rev* 2004;2.

6. Freire NB, Garcia JB, Lamy ZC. Evaluation of analgesic effect of skin-to-skin contact compared to oral glucose in preterm neonates. *Pain* 2008;139(1):28-33.

7. Carbajal R, Lenclen R, Gajdos V, et al. Cross-over trial of analgesic efficacy of glucose and pacifier in very preterm neonates during subcutaneous injections. *Pediatrics*, 2002;110(2 Pt 1):389-93.

8. Obeidat H, Kahalaf I, Callister LC, et al. Use of faciliated tucking for non-pharmacological pain management in preterm infants: a systematic review. *J Perinatal Neonatal Nurse* 2009; 23(4):372-377.

9. Axelin A, Salantera S, Kirjavainen J, et al. Oral glucose and parental holding preferable to opioid in pain management in preterm infants. *Clin J Pain* 2009:25(2):138-45.

10. Axelin A, Salantera S, Lehtonen L. 'Facilitated tucking by parents' in pain management of preterm infants-a randomized cross over trial. *Early Hum Devel* 2006; 82(4):241-7.

11. Fitzgerald M. When is an analgesicnot an analgesic? *Pain*. 2009;144:9.

12. Carmo KB, Evans N, Paradisis M. Duration of indomethacin treatment of the preterm patent ductus arteriosus as directed by echocardiography. *J Pediatr.* 2009 Dec;155(6):819-822.

13. Holsti L, Grunau RE: Initial validation of the Behavioral Indicators of Infant Pain (BIIP). *Pain*. 132, 264-272 (2007).

14. Holsti L, Grunau RE, Oberlander TF, Osiovich H.: Is it painful or not? Discriminant validity of the Behavioral Indicators of Infant Pain (BIIP) Scale. *Clin J Pain.*24, 83-88 (2008).

15. Bland JM, Altman DG. Statistical methods for assessing the agreement between two methods of clinical measurement. *Lancet* 1986;8:307-310.

16. The Observer, Base Package for Windows Reference Manual. Wageningen, The Netherlands: Noldus Information Technology, 1995.

17. Stevens B, Gibbins S. Clinical utility and clinical significance in the assessment and management of pain in vulnerable infants. *Clin Perinatol* 2002;29:459-468.

18. Stevens B, Yamada J, Beyene J, et al. Consistent management of repeated procedural pain with sucrose in preterm neonates: Is it effective and safe for repeated use over time? *Clin J Pain*. 2005;21:543-548.
